# Quantitative and Differential Analysis between *Bupleurum chinense* DC. and *Bupleurum scorzonerifolium* Willd. Using HPLC-MS and GC-MS Coupled with Multivariate Statistical Analysis

**DOI:** 10.3390/molecules28155630

**Published:** 2023-07-25

**Authors:** Zhenhuan Wang, Lu Tian, Yusheng Xiao, Mengya Zhao, Yanyan Chang, Yujiang Zhou, Shuying Liu, Huanxi Zhao, Yang Xiu

**Affiliations:** Jilin Ginseng Academy, Changchun University of Chinese Medicine, Changchun 130117, China; wangzh@ccucm.edu.cn (Z.W.); tianlulu9903@163.com (L.T.); xiaoys@ccucm.edu.cn (Y.X.); zhaomy@ccucm.edu.cn (M.Z.); changyy@ccucm.edu.cn (Y.C.); zhouyj@ccucm.edu.cn (Y.Z.); syliu@ciac.ac.cn (S.L.)

**Keywords:** *Bupleurum chinense* DC., *Bupleurum scorzonerifolium* Willd., HPLC-MS, GC-MS, multivariate statistical analysis

## Abstract

*Bupleurum chinense* DC. and *Bupleurum scorzonerifolium* Willd. have different clinical efficacies, with the former typically used to treat typhoid fever and the latter mainly used to clear liver heat. The differences in their clinical efficacy are closely related to their complex chemical composition, especially the active components. In this study, the saponins and volatile oils in two varieties of Radix Bupleuri grown in different regions were extracted and analyzed using high-performance liquid chromatography (HPLC) and gas chromatography coupled with mass spectrometry (MS), and the absolute contents of five saikosaponins were accurately quantified using an established HPLC-MS method in the multiple reaction monitoring mode. Multivariate statistical analysis was performed to reveal the difference in the active components between the two varieties. The saikosaponin content was significantly affected by variety and growing region, with all five saikosaponins being significantly higher in *Bupleurum chinense* DC. than in *Bupleurum scorzonerifolium* Willd. The results of principal component analysis and hierarchical cluster analysis show a clear distinction between the two varieties in terms of both saponins and volatile oils. Twenty-one saponins, including saikosaponin b2 and b1, and fifty-two volatile oils, including 2-tetradecyloxirane and chloromethyl cyanide, were screened and identified as differential compounds contributing to the significant difference between the two varieties. These compounds may also be responsible for the difference in clinical efficacy between *Bupleurum chinense* DC. and *Bupleurum scorzonerifolium* Willd. All the results suggest that the accumulation and diversity of active components in Radix Bupleuri are significantly affected by the variety. In contrast to previous reports, this study provides the absolute contents of five saikosaponins in Radix Bupleuri of different varieties and reduces the influence of the growing region on the analytical results by collecting samples from different regions. The results of this study may provide a reference for the identification and quality evaluation of different varieties of Radix Bupleuri.

## 1. Introduction

Radix Bupleuri, also known as “Chaihu” in Chinese, is a biennial plant belonging to the family Umbelliferae. First recorded in the *Shennong Traditional Herbal Scriptures*, it has been used as an herbal medicine for over two thousand years [1]. According to the theory of traditional Chinese medicine, Radix Bupleuri has a pungent and bitter taste, is slightly cold in nature, and is attributed to the liver, gallbladder, and lung meridians. It has been described as having the efficacies of diaphoretic, antipyretic, dispersing stagnant hepatoqi, and invigorating yang-qi effects and is commonly used in China, Japan, and other Asian countries to treat cold fever, chest tightness, rib pain, dyspepsia, menstrual disorders, and other syndromes [2].

As a commonly used Chinese herb, there are more than 60 *Bupleurum* species, varieties, and morphologies in China, such as *Bupleurum bicaule Helm*, *Bupleurum yinchowense*, *Bupleurum smithii Wolff*, and so on [3,4]. Only the dried roots of *Bupleurum chinense* DC. and *Bupleurum scorzonerifolium* Willd. are prescribed in *The Chinese Pharmacopoeia* as Radix Bupleuri, which are also known in Chinese as “Bei Chaihu” (North Radix Bupleuri, North RB) and “Nan Chaihu” (South Radix Bupleuri, South RB), respectively, due to their origin and morphology, as shown in Figure S1 [5]. They also differ in their pharmacological activities and functions, as documented in the classics of Chinese medicine, *Materia Medica Synopsis* and *Materia Medica Compendium*. North RB is typically used to treat typhoid fever, while South RB is mainly used to clear liver heat [6]. However, in most clinical practice, both are confused and used without distinction as Radix Bupleuri [7]. Therefore, it is necessary to analyze and compare the active components of North and South RB to reveal the reasons for the differences in their clinical efficacy, to improve the accuracy of clinical prescription, and to provide a reference for their quality control and evaluation.

**Figure 1:**
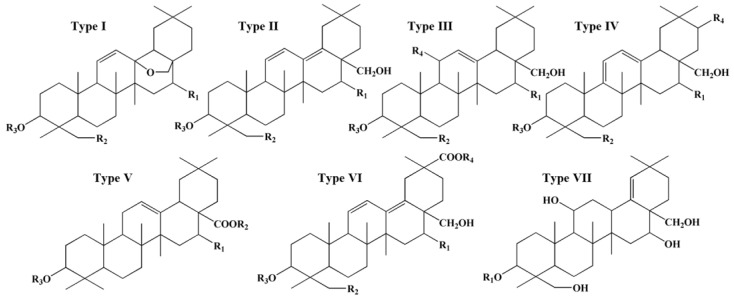
Structural types of saikosaponins.

The active components of Radix Bupleuri include saponins, volatile oils, polysaccharides, and flavonoids. Saikosaponins are generally considered the principal bioactive constituents and have anti-inflammatory, immunoregulatory, antipyretic–analgesic, and hepatoprotective effects [8,9,10,11,12]. More than 100 pentacyclic triterpenoid saikosaponins have been identified from the *Bupleurum* species and classified into seven types based on their sapogenins, namely type I of epoxy ether, type II of isocyclic diene, type III of C_12_-ene, type IV of homocyclic diene, type V of C_12_-ene-C_28_-carboxylic acid, type VI of C_30_-carboxylic acid on isocyclic diene, and type VII of C_18_-ene, as shown in Figure 1 [13]. Type I saikosaponins, represented by saikosaponin a (SSa) and saikosaponin d (SSd), are found only in *Bupleurum* plants. Their content is the highest among the seven types of saikosaponins, rendering them an important index for evaluating the quality of Radix Bupleuri [5,13].

Volatile oils are another kind of essential bioactive constituent of Radix Bupleuri. Although their total content is less than 0.2% of the root weight in Radix Bupleuri, more than 150 volatile compounds have been identified, most of which are monoterpenes and sesquiterpenes. In addition, the volatile oils in Radix Bupleuri contain a relatively higher proportion of aliphatic compounds than those found in other plants, and in particular have more alkane components, which may give them better antipyretic and anti-inflammatory effects [14]. As a result, they are widely used as the main medicinal substance in Radix Bupleuri injection for the clinical treatment of colds and fevers [15]. Altogether, saikosaponins and volatile oils, the main active components affecting the medicinal efficacy of Radix Bupleuri, are the preferred compounds for evaluating the difference between North and South RB.

Since there is a wide variety of saikosaponins and volatile oils showing diversity and similarity in structure, their rapid and accurate determination remains a major challenge [16]. The technique of coupling chromatography and mass spectrometry (MS) combines the characteristics of the high degree of separation of chromatography with the high sensitivity of MS. The detection range of high-performance liquid chromatography (HPLC)-MS and gas chromatography (GC)-MS covers almost all organic compounds and could meet the needs of the comprehensive analysis of herbal medicines [17] Collision-induced dissociation (CID)-based tandem MS is a powerful tool for the rapid qualitative analysis of target compounds through providing relative molecular mass and structural information [18]. Xia and his colleagues identified twenty-four differential metabolites from *Bupleurum marginatum* var. *stenophyllum* and *Bupleurum chinense* DC. using tandem MS analysis combined with chromatographic retention times, providing a reference for *Bupleurum* cultivar selection [19]. We also investigated the differential constituents of Radix Bupleuri cultivated in different regions using LC-MS and GC-MS [20]. A total of twenty-eight saponins and fifty-eight volatile compounds were identified by integrating fragment ion information and database retrieval. With respect to quantitative analysis, the multiple reaction monitoring (MRM) mode for MS dramatically reduces the co-elution interference by scanning predefined ion pairs and improves the accuracy and dynamic linear range of quantification. Xiu et al. employed an HPLC-MRM/MS method to accurately quantify the content of fourteen ginsenosides in cultivated ginseng and to evaluate the differences in these contents caused by origin and year of growth [21].

Recently, Qu et al. extracted and compared the metabolite compositions of different parts of wild North and South RB grown in suburban mountainous areas. They found that there were significant differences in metabolites between different tissues and speculated that the great differences in the saikosaponin content and metabolic networks were closely related to the clinical efficacy of Radix Bupleuri [6]. These results offer a highly valuable reference for guiding the clinical use of Radix Bupleuri. Limited data have been available regarding the absolute content of saikosaponins in North and South RB from different regions until now. The exact contents of saikosaponins, volatile oils, and other specific active components that affect the efficacy of the two varieties of Radix Bupleuri remain unclear and need to be discovered.

In the present study, thirty-six batches of North and South RB grown in different regions of Northwestern China were harvested and extracted to determine their saponin and volatile oil components. HPLC-MS and GC-MS combined with multivariate statistical analysis were adopted to compare and identify the differentially accumulated compounds between North and South RB. Compared to previous reports, the area in which samples were collected has been expanded. And the North and South RB samples from the same provinces were collected in as close proximity as possible to minimize differences in growing environment, hydrology, and climate. In addition, the absolute content of two major and three rare saikosaponins was determined using an established HPLC-MRM/MS quantification method to visualize the difference in saikosaponin content between the two varieties. The main aim of this study was to investigate the differences between North and South RB from the perspective of saponin and volatile oil. The findings may contribute to a deeper understanding of their differential chemical composition and the appropriate selection of *Bupleurum* varieties.

## 2. Results and Discussion

### 2.1. Differences in Saikosaponin Content between North and South RB

#### 2.1.1. Optimization of Extraction and Analysis Conditions

Since saikosaponins are prone to being converted to secondary saponins when heated, which would have consequences for the accuracy of their quantitative and statistical analysis, an ultrasonic method was used in this study to extract the primary saponins from Radix Bupleuri [22]. Ultrasonic extraction employs ultrasound to promote the penetration of saponins through the cell wall and their dissolution in the solvent. Compared to heat extraction, it is more convenient, more efficient, and more conducive to protecting unstable saponins from decomposition [22,23,24]. The extraction time, liquid-to-solid ratio, and ammonia concentration in the extraction solvent were optimized to maximize the concentration of derived saikosaponins, as described in a previous study [20]. The extracted saponins were detected in both positive and negative ion modes using an electrospray ionization (ESI) ion source. In the positive ion mode, the saponin molecules usually form charged adduct ions by binding an alkali metal ion or a hydrogen ion. This exothermic process increases their internal energy, which is distributed to all internal degrees of freedom, resulting in irregular glycosyl cross-ring cleavage and complex MS spectra during CID. Correspondingly, in the negative ion mode, the saponin molecules usually reduce their internal energy via an endothermic process of losing a proton to form an [M-H]^−^ ion or combining with a formate ion to form an [M + HCOO]^−^ ion, so that they require extra energy for CID. This process generates a limited number of fragment ions but more direct information about sapogenins and glycosidic bond cleavage and more clearly visualized spectra than its counterpart in the positive ion mode [25]. Therefore, MS data of saponins were collected in the negative ESI mode for quantitative and statistical analysis.

#### 2.1.2. Results of Method Validation

Method validation was initially performed by evaluating the linearity, limit of detection (LOD), limit of quantification (LOQ), precision, repeatability, stability, and accuracy of the developed HPLC-MRM/MS quantitative method. Five saikosaponins, namely SSa, saikosaponin c (SSc), SSd, saikosaponin e (SSe), and saikosaponin f (SSf), could be readily separated from each other within twenty minutes under the HPLC-MS conditions used, as shown in the total ion chromatogram (TIC) in Figure S2A. It is worth noting that the peak of SSd was incapable of baseline separation, but was entirely free of interference in the MRM mode (Figure S2B), which reduced the disadvantages and interferences for quantification. The calibration curves of the five saikosaponins maintained good linearity over a concentration range of two or three orders of magnitude along with correlation coefficients (R^2^) greater than 0.99, as shown in Table 1. The LOD and LOQ were calculated to be in the range of 0.006 to 0.012 μg/mL and 0.018 to 0.050 μg/mL, respectively, suggesting that the developed method is of adequate sensitivity for the detection and quantification of saikosaponins at low concentrations. As shown in Table 2, the RSDs of the repeatability test were not more than 3.20%. This indicated that the ultrasonic extraction method used could repeatedly extract saikosaponins from Radix Bupleuri in a stable manner. The extracted solutions were stable for at least twenty-four hours with a variation in saikosaponin content of less than 3.28%. The intraday and interday precision ranged from 1.62% to 3.24% and from 1.85% to 3.36%, respectively. The results of the recovery test reveal a moderate accuracy of the developed method, with mean average recoveries between 96.34% and 102.13% and a relative standard deviation (RSD) of less than 4% at the three spiked levels. In conclusion, these data validated that the HPLC-MRM/MS method fulfilled the requirements for the quantification of saikosaponins in Radix Bupleuri.

#### 2.1.3. Quantitative Analysis of Saikosaponins in North and South RB

**Figure 2:**
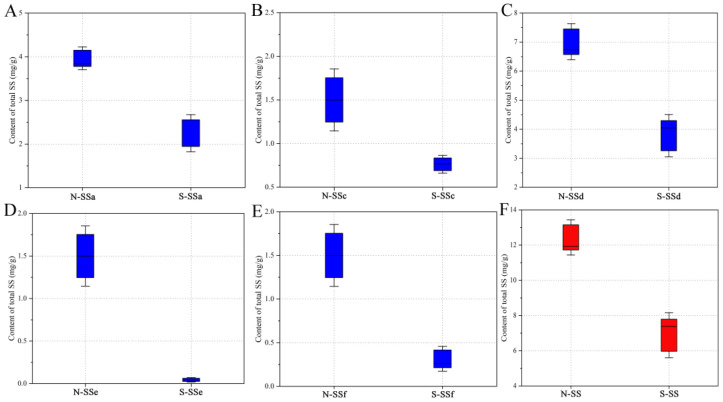
Boxplots of the content of SSa (**A**), SSc (**B**), SSd (**C**), SSe (**D**), and SSf (**E**) and their total content (**F**) in North RB and South RB.

The content of saikosaponins has been shown to vary with the region where Radix Bupleuri is cultivated [20]. Therefore, in this study, North and South RB from different regions were collected and analyzed in order to reduce the influence of the growing region on the quantitative results, which is a new addition to previous reports [6,19]. The absolute contents of the five saikosaponins in the Radix Bupleuri samples, determined using the developed HPLC-MRM/MS method, are shown in Table S1. The boxplots in Figure 2 provide a visual indication that the content of each saikosaponin was significantly lower in South RB than in North RB. The total content of the five saikosaponins in South RB was 6.882 ± 1.826 mg/g on average, whereas that in North RB was much higher at an average of 12.437 ± 1.426 mg/g. Statistics analysis was performed using independent samples *t*-test to compare the differences between North and South RB in the content of total and individual saikosaponins. There were significant differences (*p* < 0.05) for all five saikosaponins between North and South RB, indicating that the variety of Radix Bupleuri has a significant influence on the content of saikosaponins. In addition, as shown in Figure S3, the South RB grown in Yuncheng City (S-YC) and North RB grown in Sanyuan County (N-SY) had the highest contents among all the South and North RB samples, respectively. Meanwhile, the lowest contents were found in the South RB grown in Ordos City (S-OD) and the North RB grown in Baotou City (N-BT). The potential effect of variety and growing region on the content of saikosaponins was further investigated by means of analysis of variance (ANOVA), as shown in Table S2. The statistical results reveal that both variety and growing region significantly affected the saikosaponin content of Radix Bupleuri. This may be attributed to the differences in the expression of genes involved in the synthesis of saikosaponin and the metabolic pathways of saikosaponin in North and South RB, as well as the combined factors of germplasm, climate, and processing methods in different growing regions [6,26]. In summary, these results suggest that careful consideration should be given to the variety and growing region in the medical use of Radix Bupleuri.

### 2.2. Differences in Saponin and Volatile Oil Profiles between North and South RB

**Figure 3:**
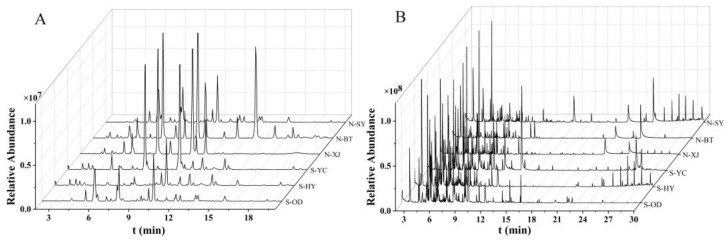
HPLC-MS analysis of saponins (**A**) and GC-MS analysis of volatile oils (**B**) in the North and South RB samples from different growing regions.

As shown in Figure 3, the TICs of the Radix Bupleuri samples, as determined via HPLC-MS and GC-MS, were complex but comparable. A total of 1129 and 1134 alcohol-soluble compounds as well as 2112 and 2104 volatile compounds were detected in the North and South RB samples, respectively. This indicates that there are different active components in different varieties of Radix Bupleuri. The analysis on the absolute content described above could provide direct insight into the differences in specific compounds. Nevertheless, as authentic standards are not available for many active components, absolute quantification could only cover a limited range of compounds. Therefore, further multivariate statistical analysis was carried out on the saponin and volatile oil profiles independently of each other to evaluate the differences between North and South RB in active components and to identify the differential compounds.

#### 2.2.1. Hierarchical Cluster Analysis (HCA)

**Figure 4:**
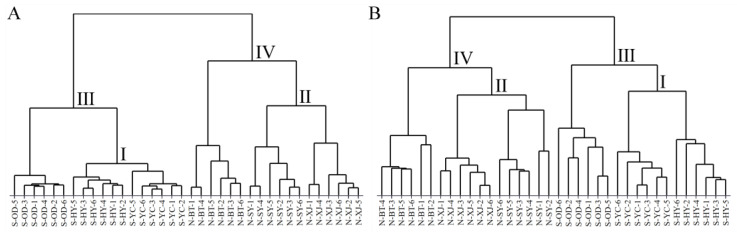
Dendrograms of North and South RB samples cultivated in different regions constructed by HCA with the HPLC-MS data sets of saponins (**A**) and the GC-MS data sets of volatile oils (**B**).

HCA is an unsupervised pattern recognition method that reveals relatively homogeneous classifications based on the similarity of samples and visualizes the proximity between samples in the dendrogram [27]. Figure 4 presents the dendrograms of HCA generated from the HPLC-MS data sets of saponin and the GC-MS data sets of volatile oil for all the Radix Bupleuri samples. It can be seen that all the samples were initially clustered between geographical replicates, indicating a good similarity between samples from the same growing region and the reliability of the HPLC-MS and GC-MS data sets. Moreover, the classification results for saponin (Figure 4A) and volatile oil (Figure 4B) were almost identical, the only difference being that the samples from Inner Mongolia were more similar to those from Shaanxi Province in terms of saponin but closer to those from Shanxi Province in terms of volatile oil. Specifically, the S-YC samples and North RB grown in Xinjiang County (N-XJ) from Shanxi Province grouped together in clusters I and II with the South RB grown in Heyang County (S-HY) and N-SY samples from Shaanxi Province, respectively, indicating the similarity of either the North RB or the South RB grown in these two provinces with respect to both saponin and volatile oil components. The S-OD and N-BT samples were subsequently classified with clusters I and II into clusters III and IV, respectively, which contained all the South and North RB samples. This suggests that there are significant differences in the saponin and volatile oil components of these two varieties of Radix Bupleuri and that the variety factor contributes more to these differences than the growing region.

#### 2.2.2. Principal Component Analysis (PCA)

**Figure 5:**
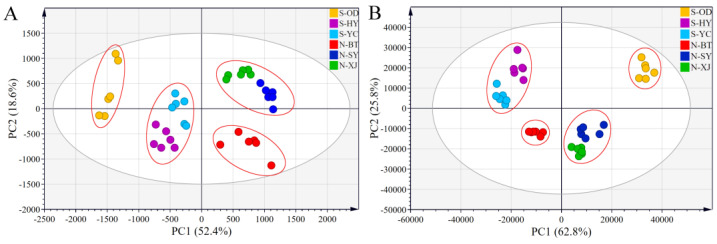
PCA score plots of North RB and South RB cultivated in different regions derived from the HPLC-MS data sets of saikosaponins (**A**) and the GC-MS data sets of volatile compounds (**B**).

PCA uses a handful of principal components to describe the correlation between multiple variables while retaining as much of the original data as possible [28]. The converted HPLC-MS and GC-MS data sets of all the North and South samples were subjected to PCA analysis. The resulting score plots for saponins and volatile oils, with each sample represented by a point, are shown in Figure 5A and 5B, respectively. All samples were free of outliers at the 95% confidence interval. The first two principal components (PC1 and PC2) accounted for 52.4% and 18.6%, that is 71.0%, of the total variability in the original HPLC-MS data sets (Figure 5A), while they accounted for 88.6% in the GC-MS data sets (Figure 5B).

Whether for saponins or volatile oils, all the Radix Bupleuri samples could be clearly divided into two regions by the origin of the PC1. The North RB samples were located in the positive region to the right of the origin, while the South RB samples were located in the negative region to the left of the origin. These results confirm that there are significant differences in the active components between different varieties of Radix Bupleuri, even for samples grown in the same province, and that the variety is a vital factor affecting the quality of Radix Bupleuri. *The Chinese Pharmacopoeia* described the oleaginous smell of South RB, implying its characteristic volatile composition and explaining the apparent separation of North and South RB samples in Figure 5B [5]. There was an obvious grouping tendency among samples from the same growing region, indicating that these herbs were of homogeneous quality. Moreover, samples from the Shanxi and Shaanxi Provinces were closer together, whereas samples from Inner Mongolia were far apart from them. This may be explained by the geographical proximity of the Yuncheng and Xinjiang Counties in Shanxi Province and the Heyang and Sanyuan Counties in Shaanxi Province. Their similar climate, natural environment, and germplasm resources could result in similar characteristics of the cultivated North and South RB samples. HCA and PCA allowed qualitative comparison and effective differentiation between samples of different varieties of Radix Bupleuri. The combination of approximate results from the two statistical analysis methods provided some evidence that the intrinsic quality of North and South RB differed considerably, and that the dynamic accumulation of active components in different varieties of Radix Bupleuri contributed to their different efficacy.

#### 2.2.3. Partial Least-Squares–Discriminant Analysis (PLS-DA)

**Figure 6:**
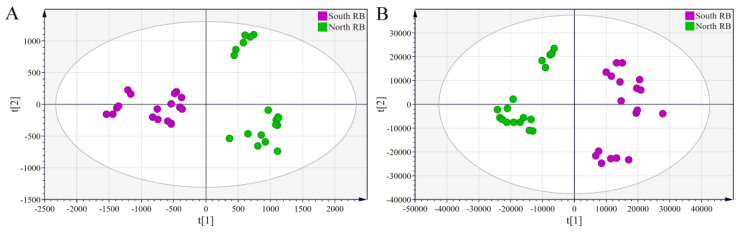
PLS-DA scores plots derived from the HPLC-MS data sets of saponin compounds (**A**) and the GC-MS data sets of volatile oils (**B**) in North and South RB.

PLS-DA is a supervised pattern recognition technique. It reduces intragroup error and random error that is not relevant to the study objective by setting mandatory groups, allowing more accurate analysis of information about the variability of characteristics between samples. PLS-DA models were developed for saponin and volatile oil using the converted HPLC-MS and GC-MS data sets, respectively. The parameters of the PLS-DA model were R^2^ = 0.983 and Q^2^ = 0.918 for saponin (Figure 6A) and R^2^ = 0.976 and Q^2^ = 0.952 for volatile oil (Figure 6B). R^2^ and Q^2^ represent the ability of the model to describe the data and the ability to predict new observed data, respectively. The closer they are to 1.0, the better the model fits and predicts [29]. Therefore, both PLS-DA models developed have a good fit and predictive ability. Furthermore, the results of 200 random permutation tests of both models are shown in Figure S4. It can be seen that all permuted R^2^ and Q^2^ were smaller than the original values of their models, and the regression line for permuted Q^2^ had a negative intercept on the *y*-axis, suggesting that the models were not over-fitted.

**Figure 7:**
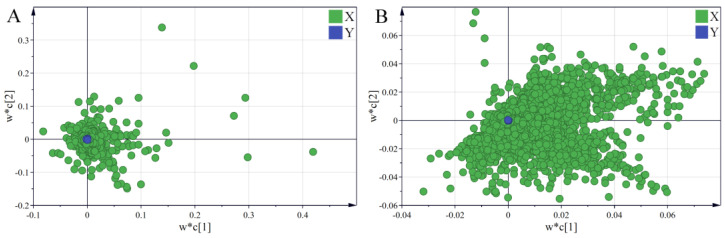
Loading plots of PLS-DA models derived from the HPLC-MS data sets of saponin compounds (**A**) and the GC-MS data sets of volatile oils (**B**) in North and South RB.

As shown in Figure 6, a significant segregation occurred between the North and South RB samples, indicating the presence of significantly different saponin and volatile oil compounds. The samples grown in the same region still showed a high degree of similarity despite being grouped on the basis of the varieties, suggesting that the growing region has a considerable impact on the quality of Radix Bupleuri. Variable importance for the projection (VIP) values of saponins and volatile oils were further calculated and ranked from the respective PLS-DA models. The VIP represents the importance of the variable in the explanation of the data sets and in relation to the grouping [30]. Combined with the loading plots (Figure 7), VIP > 1 was used as an indicator to screen for differential saponin and volatile oil components in North and South RB. Twenty-one differential saponins and fifty-two differential volatile oils were identified. The saponins were identified via tandem MS and comparison with literature data.

**Figure 8:**
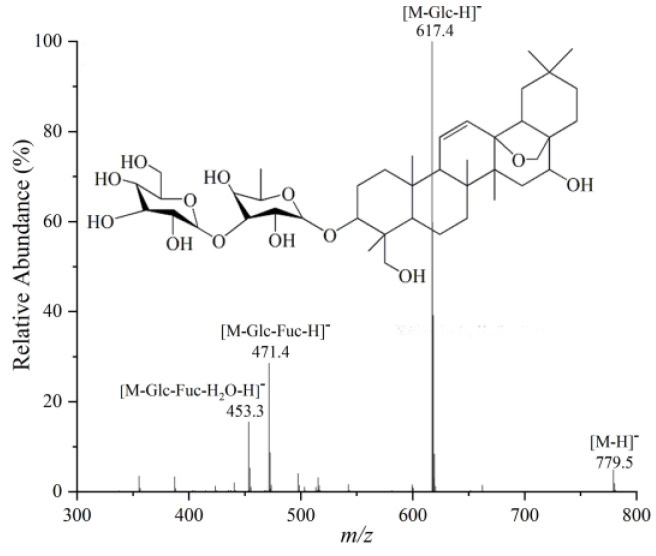
MS/MS spectrum of SSa.

Using the example of SSa, its retention time was 6.40 min and its relative molecular mass was calculated to be 780.5, from which its molecular formula was deduced to be C_42_H_68_O_13_. As can be seen from its tandem mass spectrum in Figure 8, the [M − H]^−^ ion at *m*/*z* 779.5 sequentially lost one molecule of the glucose substituent (162 Da), one molecule of the fucose substituent (146 Da) and one molecule of water (18 Da) to give the product ions at *m*/*z* 617.4, *m*/*z* 471.4, and *m*/*z* 453.3, which were assigned to [M – Glc − H]^−^, [M – Glc – Fuc − H]^−^, and [M – Glc – Fuc − H_2_O − H]^−^ ions, respectively. The ion at *m*/*z* 453.3 is the characteristic sapogenin ion of type I saikosaponin, demonstrating that this compound is a type I saikosaponin consisting of one molecule of fucose and one molecule of glucose. Comparison with its retention time and tandem mass spectrum reported in the literature allowed the compound to be identified as SSa. On the other hand, the volatile oils were identified by searching the NIST database for mass spectra of the compounds. As shown in Table 3 and Table 4, the identified differential saponins and volatile oil compounds were mainly triterpene saponins and their derivatives, aliphatic compounds, alcohols, and aldehydes. They showed significant differences (*p* < 0.05) between the North and South RB samples, as revealed by *t*-tests.

Overall, in contrast to previous work, the present study concentrated on analyzing the differences in saponins and volatile oils between North and South RB from different regions. The wide range of collection areas was conducive to reducing the effect of the growing region on the analytical results. The absolute content of five saikosaponins was determined in thirty-six batches of Radix Bupleuri samples. The two varieties showed significant differences in the active components, and a total of seventy-three differential compounds were identified. A detailed comparison of this work with the reported work is given in Table S3.

## 3. Materials and Methods

### 3.1. Samples and Chemicals

Eighteen batches of two-year-old *Bupleurum chinense* DC. (North RB) and eighteen batches of two-year-old *Bupleurum scorzonerifolium* Willd. (South RB) were collected in October and November 2021 from Shaanxi Province, Shanxi Province, and the Inner Mongolia Autonomous Region in China. Detailed information on these samples is given in Table 5. Their botanical origins were authenticated by Professor Jiyu Gong of Changchun University of Chinese Medicine, China. Five commercially available standards, SSa, SSc, SSd, SSe, and SSf, with a purity of more than 98%, were purchased from Shanghai Yuanye Biological Technology Co., Ltd. (Shanghai, China). Chromatographic-grade methanol, acetonitrile, formic acid, and *n*-hexane were purchased from Tedia Company, Inc. (Fairfield, CT, USA). Analytical-grade methanol, ammonia, and *n*-butanol were acquired from Beijing Chemical Industry Group Co, Ltd. (Beijing, China). Ultrapure water was obtained from a Milli-Q water purification system manufactured by Millipore Corporation (Burlington, MA, USA).

### 3.2. Sample Extraction

#### 3.2.1. Extraction of Saponins

Extraction was performed under optimized conditions, as described in the previous study [20]. Radix Bupleuri samples were washed, dried, pulverized, and passed through a 40-mesh sieve. A total of 0.5 g of sample powder was mixed with 25 mL of 15% ammonia–methanol solution and was extracted via sonication for 60 min at room temperature, followed by filtration. The filtrates were evaporated to dryness in a water bath and redissolved in 5 mL of *n*-butanol, followed by centrifugation at 7000 rpm for 5 min. The supernatant was evaporated to dryness again and redissolved in 5 mL of chromatographic-grade methanol prior to HPLC-MS analysis.

#### 3.2.2. Extraction of Volatile Oils

An amount of 25.0 g of Radix Bupleuri powder was placed in a steam distillation apparatus with 250 mL of ultrapure water and soaked for 12 h. The graduated tube was filled with ultrapure water and sealed with 2 mL of *n*-hexane. Extraction was carried out via steam distillation for 8 h. The *n*-hexane layer was then collected, dehydrated with anhydrous magnesium sulfate, and diluted to 5 mL with *n*-hexane before GC-MS analysis. The extraction method is the same as previously reported [20].

### 3.3. Instrument Conditions

#### 3.3.1. HPLC-MS Conditions

Saponin extracts were analyzed using an Ultimate 3000 HPLC coupled with a TSQ Endura triple-quadrupole MS (Thermo Fisher Scientific, San Jose, CA, USA) equipped with a Thermo Syncronis C_18_ column (100 mm × 2.1 mm, 1.7 μm). The mobile phase consisted of ultrapure water with 0.1% (*v*/*v*) formic acid (A) and acetonitrile (B). The gradient elution program was carried out as follows: 35–90% A for 0–20 min, 90–35% A for 20–20.5 min, and 35% A for 20.5–25 min at a flow rate of 0.2 mL/min. The column temperature was set at 25 °C and the injection volume was 2 µL. The effluent was delivered to the MS via the ESI ion source with the sheath gas, auxiliary gas, and sweep gas set to 38, 11, and 1 arbitrary unit, respectively. The spray voltage, ion transfer tube temperature, and vaporizer temperature were set at −2500 V, 329 °C, and 296 °C, respectively. Ultrapure argon was introduced as the collision gas, and the collision energy was between 15 and 45 V. MS was operated in full-scan, product ion scan, and MRM modes for the differential, qualitative, and quantitative analysis of the saponins, respectively.

#### 3.3.2. GC-MS Conditions

Volatile oil extracts were analyzed using a Trace 1310 GC apparatus coupled with a TSQ 8000 triple-quadrupole MS (Thermo Fisher Scientific, San Jose, CA, USA) equipped with a DB-5MS capillary column (30 m × 0.25 mm, 0.25 μm, Agilent Technologies, Folsom, CA, USA). Ultrapure helium was used as the carrier gas at a constant flow rate of 1.2 mL/min. The injection port was kept at 250 °C in split mode. The injection volume was 2 µL. The initial oven temperature was held at 60 °C for 2 min, and then increased to 140 °C at 15 °C/min and held for 2 min, increased to 180 °C at 5 °C/min and held for 3 min, and increased to 250 °C at 10 °C/min and held for 10 min. The effluent was delivered to the electron impact ion source at 230 °C via an MS transfer line at 230 °C. Ultrapure argon was used as the collision gas, and the collision energy was between 5 and 20 eV. Data were collected in full-scan mode for the profile analysis and in product ion mode for the assisted identification of volatile oils.

### 3.4. Qualitative and Quantitative Analysis

In general, saponins were identified via resolution of the structural information obtained using tandem MS analysis and the retention time. The relative molecular weight and molecular formula were calculated from the quasi-molecular ion pair of [M − H]^−^ and [M + HCOO]^−^ ions in negative ion mode. Information on the type and number of glycosyl substituents and sapogenins was collected from the fragment ions generated by CID and then used to infer the structures, which were further verified through comparison with fragmentation data in the literature. On the other hand, the identification of volatile oils was performed by retrieving their background-subtracted MS spectra in the NIST 14 database, and was supplemented with tandem MS analysis.

The absolute contents of SSa, SSc, SSd, SSe, and SSf in Radix Bupleuri were determined using an established HPLC-MRM/MS method. Saikosaponin standards were precisely weighed and dissolved in HPLC-grade methanol to prepare individual and mixed standard stock solutions, which were used to optimize MRM parameters and to construct calibration curves, respectively. The individual standard solution was injected directly and continuously into the ion source via an infusion pump. It was examined using a built-in program to acquire the optimized MRM parameters, including the precursor ion, the two most intense product ions appearing at most of the collision energies applied, and the corresponding collision energy (Table 6). All samples and diluted mixed standards were detected in MRM mode by scanning the optimized parameters of the five saikosaponins. The absolute content of each saikosaponin was then calculated through the external standard method based on the automatically integrated peak areas in the total ion chromatography using Xcalibur software (version 2.2). Each sample was quantified with three replicates, and the contents are expressed as mean and standard deviation.

### 3.5. Method Validation

The standard curve equation for saikosaponin was constructed by linearly fitting the peak areas of seven standard solutions at different concentrations, and its linearity was evaluated with the linear regression coefficient (R^2^). Saikosaponin contents detected at signal-to-noise ratios of 3 and 10 were determined as LOD and LOQ, respectively. Precision was evaluated by interday and intraday precision, which were defined as the variation in the saikosaponin content of a sample measured six times in one day and measured three times in one day for three consecutive days, respectively. The variation was expressed as RSD of saikosaponin content. Stability was validated by analyzing the variation in the content of each saikosaponin in a sample at 4 h intervals over 24 h. A sample of Radix Bupleuri powder was divided into six equal portions to individually determine the content of the five saikosaponins contained therein, the variation in which was expressed as the repeatability of the extraction method. Accuracy was assessed using sample recovery tests. The Radix Bupleuri powder was added with saikosaponin standards of 80%, 100%, and 120% of the original content and was then extracted to calculate the recoveries using the following equation: recovery (%) = 100 × (found amount − original amount)/added amount. The test was repeated three times for each saikosaponin at each spiked level.

### 3.6. Data Processing and Statistical Analysis

HPLC-MS and GC-MS data acquisition was performed with Xcalibur software (version 2.2 SP1.48, Thermo Fisher Scientific, San Jose, CA, USA). The acquired data profiles were processed using SIEVE software (version 2.1, Thermo Fisher Scientific Inc., San Jose, CA, USA) for peak matching, peak alignment, and peak area normalization, followed by removal of isotopic peaks, repeated peaks, and missing values greater than 80% before statistical analysis. The resulting data were then subjected to SIMCA-P software (version 14.1, Umetrics, Umeå, Sweden) for PCA, HCA, and PLS-DA. Independent samples *t*-test, ANOVA, and boxplot analysis were performed to evaluate the difference in saikosaponin content between North and South RB using OriginPro software (version 9.1, OriginLab Corporation, Northampton, MA, USA). The level of statistical significance was set at *p* < 0.05.

## 4. Conclusions

The complex and unique chemical compositions in North and South RB greatly affect their clinical efficacy. In the current study, saponin and volatile oil components were extracted from North and South RB grown in different regions, and the differential compounds and absolute contents of five saikosaponins were determined using HPLC-MS and GC-MS combined with multivariate statistical analysis. The results of the statistical analysis show that the content of saikosaponins was significantly influenced by variety and growing region, with the content of saikosaponins in North RB being significantly higher than that in South RB. Radix Bupleuri samples of the same variety and growing region were remarkably similar in their active components. Significant differences were found between the active components of the two varieties whether they were grown in the same region or not. Twenty-one saponin and fifty-two volatile oil compounds were identified as differential compounds, most of which were triterpene saponins and their derivatives and aliphatic, alcoholic, and aldehydic compounds. They contributed significantly to the differences between North and South RB and may be the main active components responsible for the differences in their clinical efficacy.

The current study demonstrates the differences between North and South RB in terms of saponins and volatile oils. It will benefit from pathway enrichment analyses of the identified differential compounds to provide more insight into the origin of these differences between the two varieties, as reported in previous work. In addition, due to the limitations of the available standard references, quantitative analyses were performed for only five saponins and without the volatile oil components. As pharmacological activity may depend on a number of different chemical compounds, the current study could therefore provide a reference for research on the differential composition of different varieties of Radix Bupleuri, but not a direct relationship between constituent content and pharmacological activity. The correlation between the variety and the content of differential compound and the efficacy of Radix Bupleuri still need to be verified with further pharmacological studies.

## Figures and Tables

**Table 1:** Calibration curve, R^2^, and linear range of the five saikosaponins detected using the HPLC-MRM/MS method.

Saikosaponin	Calibration Curve	R^2^	Linear Range (μg/mL)	LOD (μg/mL)	LOQ (μg/mL)
SSa	y = 117,587 + 5695.15x	0.9959	0.1–100.0	0.008	0.025
SSc	y = 102,954 + 8278.13x	0.9957	0.05–10.0	0.007	0.020
SSd	y = 97,594 + 5994.61x	0.9925	0.1–100.0	0.012	0.050
SSe	y = 232,657 + 12,748.1x	0.9944	0.03–3.0	0.008	0.018
SSf	y = 98,469.8 + 8989.99x	0.9966	0.05–10.0	0.006	0.020

**Table 2:** Precision, repeatability, stability, and recovery of the five saikosaponins detected using the HPLC-MRM/MS method.

Saikosaponin	Intraday Precision RSD (*n* = 9, %)	Interday Precision RSD (*n* = 6, %)	Repeatability RSD (*n* = 6, %)	Stability RSD (*n* = 7, %)	Recovery (%)/RSD (*n* = 3, %)		
					80%	100%	120%
SSa	2.89	1.85	2.12	2.03	98.62/1.68	99.68/1.98	101.13/2.09
SSc	1.96	2.06	1.81	1.65	98.91/1.85	101.34/1.47	97.65/1.98
SSd	1.62	2.16	1.26	1.46	101.03/1.42	99.85/1.25	98.73/1.93
SSe	3.24	3.36	3.13	3.28	101.07/1.35	98.90/2.19	98.46/1.29
SSf	3.15	3.03	3.20	2.92	97.83/2.13	102.13/1.98	96.34/2.64

**Table 3:** Information on the differential saponin compounds between North and South RB.

No.	t_R_ (min)	VIP	Compound Name	[M − H]^−^ Ion (*m*/*z*)	Fragment Ions (*m*/*z*)	Molecular Formula	References
1	3.63	1.6	SSs	941.5	941.5, 780.5, 617.4, 454.4	C_48_H_78_O_18_	[2]
2	3.98	1.1	isorhamnetin-3-*O*-β-D glucoside	477.4	477.4, 317.3	C_22_H_22_O_12_	[2]
3	4.24	3.7	SSt (Bupleuroside IX)	795.5	795.5, 633.4, 557.4, 487.4, 455.4, 407.4	C_42_H_68_O_14_	[20]
4	4.64	1.2	SSi	925.5	779.5, 763.5, 617.4, 455.4	C_48_H_78_O_17_	[31]
5	4.72	2.6	isomer of hydroxy SSc	943.5	943.5, 781.5, 797.5, 635.4, 617.4, 599.4, 455.4	C_48_H_80_O_18_	[31]
6	5.67	3.6	malonyl-acetyl-hydroxy SSa or d	883.5	797.5, 635.4, 559.4	C_45_H_72_O_17_	[2]
7	6.69	1.7	SSc	925.5	925.5, 779.5, 763.5, 617.4, 455.4	C_48_H_78_O_17_	[20]
8	7.56	3.4	SSf	927.5	927.5, 781.5, 765.5, 619.4, 457.4	C_48_H_80_O_17_	[20]
9	9.08	1.1	SSb4	811.5	811.5, 649.4, 503.4	C_43_H_72_O_14_	[20]
10	9.36	1.9	2″-*O*-acetyl SSb3	853.5	853.5, 811.5, 793.5, 649.4, 471.4, 439.4	C_45_H_74_O_15_	[31]
11	10.33	1.6	SSa	779.5	779.5, 617.4, 471.4, 453.4	C_42_H_68_O_13_	[20]
12	10.5	18.3	SSb2	779.5	779.5, 617.4, 541.4, 471.4	C_42_H_68_O_13_	[19]
13	11.43	2.7	2″-*O*-acetyl SSa	821.5	821.5, 779.5, 761.5, 617.4, 471.4	C_44_H_70_O_14_	[13]
14	11.66	1.5	malonyl-SSa	865.5	865.5, 821.5, 779.5, 761.5, 617.4, 471.4	C_45_H_70_O_16_	[2]
15	11.85	18.5	SSb1	779.5	779.5, 617.4, 471.4	C_42_H_68_O_13_	[13]
16	12.75	3.4	SSe	779.5	763.5, 601.54 455.4	C_42_H_68_O_13_	[13]
17	14.14	2.7	SSd	779.5	779.5, 617.4, 471.4	C_42_H_68_O_13_	[20]
18	14.82	2.7	6″-*O*-acetyl-SSa	821.5	821.5, 779.5, 761.5, 617.4, 471.4	C_44_H_70_O_14_	[13]
19	15.51	1.5	malonyl-SSd	865.5	865.5, 821.5, 779.5, 761.5, 617.4, 471.4	C_45_H_70_O_16_	[2]
20	17.11	1.5	malonyl-SSb2	865.5	821.5, 779.5, 617.4, 541.4	C_42_H_68_O_13_	[31]
21	20.9	2.8	prosaikogenin g	617.5	617.4, 541.4, 471.4, 439.4	C_36_H_58_O_8_	[31]

**Table 4:** Information on the differential volatile oil compounds from North RB and South RB cultivated in different regions analyzed using GC-MS.

No.	t_R_ (min)	VIP	Compound Name	M^+^• Ion (*m*/*z*)	Molecular Formula
1	2.55	1.8	1,3,5-cycloheptatriene	92.1	C_7_H_8_
2	4.03	2.3	2-butene	56.1	C_4_H_8_
3	4.03	1.8	methylsuccinic anhydride	114.1	C_5_H_6_O_3_
4	4.07	1.5	pyrrolidine	71.1	C_4_H_9_N
5	4.4	1.2	cyclohexa-1,4-diene	152.3	C_10_H_16_O
6	4.89	1.6	phenacyl thiocyanate	177.2	C_9_H_7_NOS
7	4.91	1.1	(bromomethyl) cyclopropane	135.0	C_4_H_7_Br
8	4.98	1.8	(*Z*)-2-pentene	70.1	C_5_H_10_
9	5.01	1.5	1,7,7-trimethylbicyclo heptane-2,3-dione	166.2	C_10_H_14_O_2_
10	5.06	1.6	*n*-methylhexan-1-amine	115.2	C_7_H_17_N
11	5.29	1.5	2,3,3-trimethyl-1-butene	98.2	C_7_H_14_
12	5.38	2.5	hexanoic acid	116.2	C_6_H_12_O_2_
13	5.41	1.4	bis(chloromethyl) ether	115.0	C_2_H_4_C_l2_O
14	5.46	1.8	2,2,4,4,6-pentamethylheptane	170.3	C_12_H_26_
15	5.49	1.9	1-methyl-2-(1-methylethyl)-benzene	134.2	C_10_H_14_
16	5.81	1.1	4,5-dihydro-2-pentalenone	120.2	C_8_H_8_O
17	6.05	2.7	hexanoic acid,1-cyclopentylethyl ester	212.3	C_13_H_24_O_2_
18	6.05	2.0	2-methyl-2-nitro-1-propanol	119.1	C_4_H_9_NO_3_
19	6.05	2.7	*trans*-2-undecenoic acid	184.3	C_11_H_20_O_2_
20	6.09	1.3	dimethyl sulfate	126.1	C_2_H_6_O_4_S
21	6.1	1.1	acetophenone	120.2	C_8_H_8_O
22	6.12	3.4	chloromethyl cyanide	75.5	C_2_H_2_ClN
23	6.17	3.5	2-tetradecyloxirane	240.4	C_16_H_32_O
24	6.86	1.6	undecanoic acid,	184.3	C_11_H_20_O_2_
25	6.86	1.2	isopinocarveol	152.2	C_10_H_16_O
26	7.21	3.1	octanoic acid	144.2	C_8_H_16_O_2_
27	7.21	3.1	isopulegol	154.3	C_10_H_18_O
28	7.61	1.3	didodecyl phthalate	502.8	C_32_H_54_O_4_
29	8.04	2.7	geosmin	182.3	C_12_H_22_O
30	8.09	1.2	dibutyl methanephosphonate	208.2	C_9_H_21_O_3_P
31	8.11	1.6	1,2-dimethylcyclohexane	112.2	C_8_H_16_
32	8.68	1.6	5-isopropyl-3-methylphenol	150.2	C_10_H_14_O
33	9.41	1.4	5-propyldihydro-2-furanone	128.2	C_7_H_12_O_2_
34	9.80	1.5	lauraldehyde	184.3	C_12_H_24_O
35	10.81	1.8	chloral hydrate	165.4	C_2_H_3_C_l3_O_2_
36	11.11	2.2	hexyl octyl ether	214.4	C_14_H_30_O
37	11.11	2.2	(*Z*)-dodec-5-enol	184.3	C_12_H_24_O
38	11.8	2.2	(11*Z*)-11-hexadecenoic acid	254.4	C_16_H_30_O_2_
39	11.81	2.5	2-hexyl-(1*R*,2*R*)-cyclopropaneacetic acid	184.3	C_11_H_20_O_2_
40	12.83	2.3	10-heneicosene	294.6	C_21_H_42_
41	12.83	2.3	10-methyldodec-2-en-4-olide	210.3	C_13_H_22_O_2_
42	12.86	2.8	(2*Z*,6*E*)-farnesyl acetate	264.4	C_17_H_28_O_2_
43	24.06	2.0	methyl heptadecadienoate	280.0	C_18_H_32_O_2_
44	24.06	2.0	ethyl oleate	310.5	C_20_H_38_O_2_
45	24.07	2.1	2-chlorocyclohexanol	134.6	C_6_H_11_ClO
46	24.26	2.4	(8*Z*,10*Z*)-hexadecadien-1-ol acetate	238.4	C_16_H_30_O
47	24.27	3.0	2-methyl-octadecane	268.5	C_19_H_40_
48	24.3	2.6	methyl octadeca-9,12-dienoate	294.5	C_19_H_34_O_2_
49	29.74	2.9	hentriacontane	436.8	C_31_H_64_
50	31.55	3.1	cyclopentadecanol	226.4	C_15_H_30_O
51	32.59	2.1	decane	142.2	C_10_H_22_
52	37.07	3.3	digitoxin	764.9	C_41_H_64_O_13_

**Table 5:** Sources of the North and South RB samples.

Growing Region	Administrative District	Variety	Abbreviation
Xinjiang County	Shanxi	North RB	N-XJ
Sanyuan County	Shaanxi	North RB	N-SY
Baotou City	Inner Mongolia	North RB	N-BT
Yuncheng City	Shanxi	South RB	S-YC
Heyang County	Shaanxi	South RB	S-HY
Ordos City	Inner Mongolia	South RB	S-OD

**Table 6:** MRM parameters of five saikosaponins.

Saikosaponin	t_R_ (min)	Precursor Ion (*m*/*z*)	Product Ion I (*m*/*z*)/ Collision Energy (eV)	Product Ion II (*m*/*z*)/ Collision Energy (eV)
SSc	6.69	971.5	779.3/38.9	925.4/23.7
SSf	7.56	973.5	781.3/39.9	927.4/23.4
SSa	10.33	779.5	439.6/52.0	617.3/32.2
SSe	12.75	809.5	601.3/33.0	763.3/21.3
SSd	14.14	779.5	439.2/55.0	617.3/33.1

## Data Availability

Not applicable.

## Supplementary Materials

The following supporting information can be downloaded at: https://www.mdpi.com/article/10.3390/molecules28155630/s1. Figure S1: pictures of *Bupleurum chinense* DC. (A) and *Bupleurum scorzonerifolium* Willd. (B); Figure S2: total ion chromatograms of extracted saponins detected via HPLC-MS in full-scan (A) and MRM mode (B); Figure S3: boxplots of the total saikosaponin contents in the South (A) and North RB samples (B) from different growing regions; Figure S4: permutation tests of PLS-DA models derived from the HPLC-MS data sets of saikosaponins (A) and the GC-MS data sets of volatile oils (B) in the Radix Bupleuri samples; Table S1: the absolute contents of the five and total saikosaponins in the North and South RB samples; Table S2: ANOVA of the effects of variety and growing region on the contents of the five saikosaponins; Table S3: comparison of current and reported work.
