# Supplementary material for: Quantitative and Differential Analysis between Bupleurum chinense DC. and Bupleurum scorzonerifolium Willd. Using HPLC-MS and GC-MS Coupled with Multivariate Statistical Analysis
Source: Molecules. 2023 Jul 25;28(15):5630. doi: 10.3390/molecules28155630 (PMC10419597; doi:10.3390/molecules28155630)
Supplement: Supplementary file 1 [file molecules-28-05630-s001.zip › molecules-2504211-supplementary.pdf]

Supplementary Materials

Quantitative and Differential Analysis between *Bupleurum chinense* DC. and *Bupleurum scorzonerifolium* Willd. Using High-Performance Liquid Chromatography-Mass Spectrometry and Gas Chromatography-Mass Spectrometry Coupled with Multivariate Statistical Analysis

Zhenhuan Wang †, Lu Tian †, Yusheng Xiao, Mengya Zhao, Yanyan Chang, Yujiang Zhou, Shuying Liu, Huanxi Zhao * and Yang Xiu *

Jilin Ginseng Academy, Changchun University of Chinese Medicine, Changchun 130117, China; wangzh@ccucm.edu.cn (Z.W.); tianlulu9903@163.com (L.T.); xiaoys@ccucm.edu.cn (Y.X.); zhaomy@ccucm.edu.cn (M.Z.); changyy@ccucm.edu.cn (Y.C.) zhouyj@ccucm.edu.cn (Y.Z.); syliu@ciac.ac.cn (S.L.)

* Correspondence: zhaohx@ccucm.edu.cn (H.Z.); xiuyang@ccucm.edu.cn (Y.X.); Tel.: +86-431-86763993 (H.Z.); +86-431-86763985 (Y.X.)

† These authors contributed equally to this work.

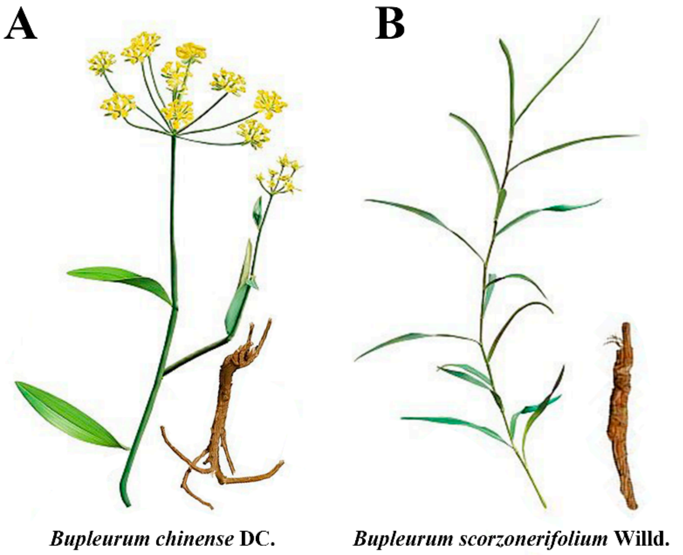

Figure S1. The pictures of *Bupleurum chinense* DC. (A) and *Bupleurum scorzonerifolium* Willd. (B).

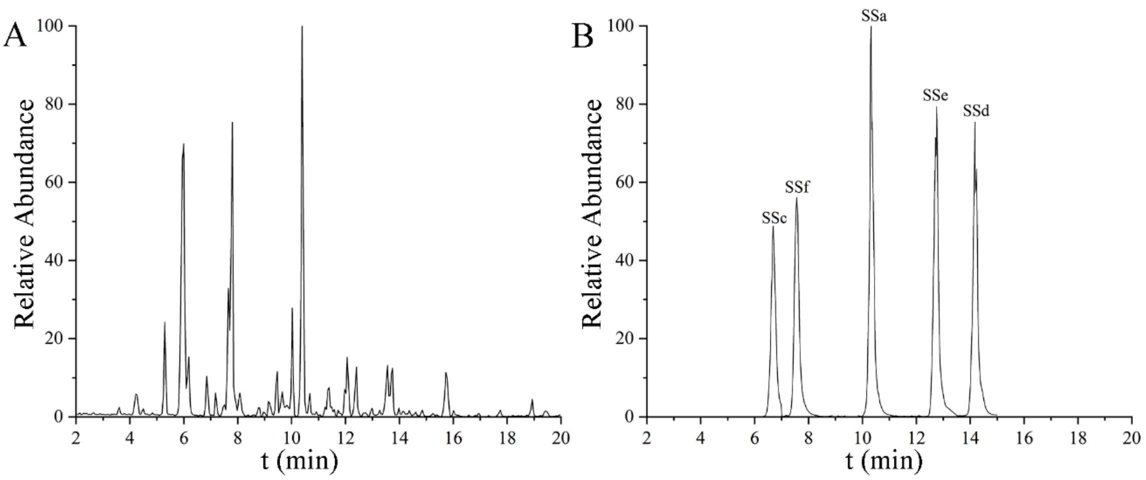

Figure S2. Total ion chromatograms of extracted saponins detected by HPLC-MS in full scan (A) and MRM mode (B).

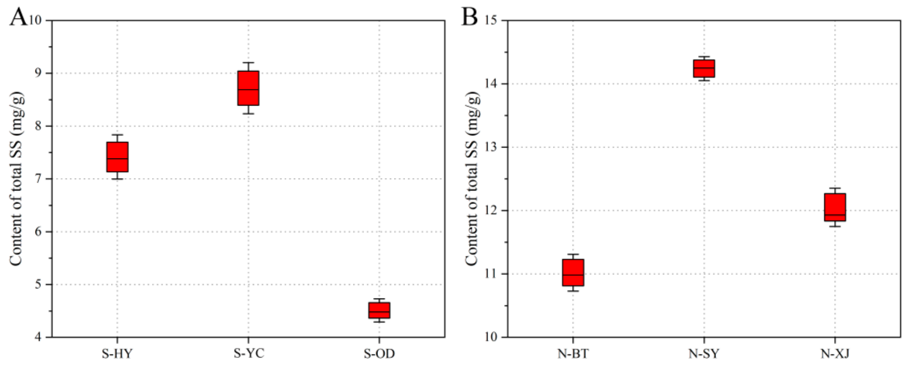

Figure S3. Boxplots of the total saikosaponin contents in the South (A) and North (B) RB samples from different growing regions.

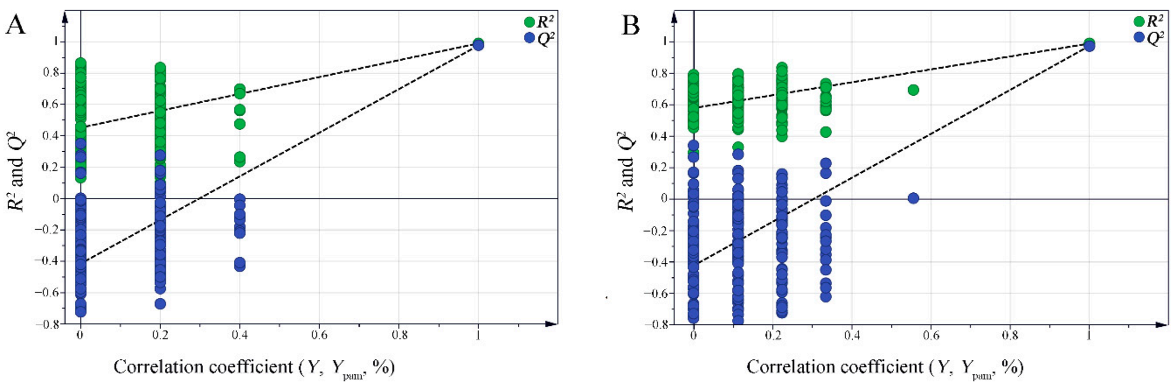

Figure S4. Permutation tests of PLS-DA models derived from the HPLC-MS data sets of saikosaponins (A) and the GC-MS data sets of volatile oils (B) in the Radix Bupleuri samples.

Table S1. The absolute contents of the five and total saikosaponins in the North and South RB samples.

No.	Content of SS[(1)] (μg/mg)					
	SSa	SSc	SSd	SSe	SSf	Total SS
N-BT-1[(2)]	3.523±0.008	0.820±0.006	5.853±0.007	0.048±0.004	0.334±0.011	10.578±0.047
N-BT-2	3.572±0.023	0.829±0.002	6.070±0.029	0.036±0.013	0.398±0.009	10.904±0.050
N-BT-3	3.632±0.012	0.834±0.010	6.233±0.009	0.055±0.007	0.308±0.006	11.063±0.016
N-BT-4	3.568±0.009	0.821±0.031	5.877±0.004	0.057±0.017	0.328±0.007	10.652±0.023
N-BT-5	3.710±0.012	0.815±0.043	6.314±0.023	0.033±0.008	0.349±0.009	11.221±0.011
N-BT-6	3.714±0.012	0.845±0.022	6.730±0.004	0.042±0.002	0.378±0.012	11.710±0.021
N-SY-1	4.564±0.051	0.935±0.066	8.076±0.046	0.095±0.003	0.619±0.007	14.290±0.056
N-SY-2	4.639±0.064	0.962±0.005	7.873±0.034	0.107±0.009	0.628±0.006	14.209±0.087
N-SY-3	4.378±0.039	0.955±0.005	8.470±0.035	0.094±0.011	0.638±0.009	14.535±0.077
N-SY-4	4.589±0.054	0.983±0.009	8.164±0.067	0.141±0.002	0.651±0.023	14.528±0.022
N-SY-5	4.219±0.006	0.968±0.015	7.971±0.008	0.111±0.013	0.613±0.029	13.882±0.052
N-SY-6	4.182±0.051	0.951±0.013	8.097±0.005	0.105±0.002	0.664±0.019	13.998±0.067
N-XJ-1	3.816±0.028	0.892±0.011	6.514±0.006	0.079±0.007	0.522±0.022	11.823±0.039
N-XJ-2	3.670±0.039	0.891±0.009	6.888±0.041	0.055±0.009	0.503±0.002	12.037±0.022
N-XJ-3	3.822±0.077	0.911±0.024	6.469±0.019	0.057±0.006	0.515±0.008	11.775±0.091
N-XJ-4	3.956±0.008	0.922±0.003	7.452±0.014	0.062±0.008	0.469±0.009	12.866±0.068
N-XJ-5	3.914±0.009	0.894±0.005	6.739±0.091	0.091±0.001	0.478±0.007	12.116±0.011
N-XJ-6	3.855±0.042	0.860±0.029	6.418±0.006	0.066±0.007	0.494±0.004	11.694±0.041
S-HY-1[(3)]	2.499±0.049	0.611±0.016	3.851±0.009	0.024±0.002	0.146±0.014	7.118±0.087
S-HY-2	2.533±0.023	0.614±0.007	3.993±0.047	0.020±0.002	0.174±0.007	7.327±0.059
S-HY-3	2.576±0.025	0.611±0.003	4.101±0.079	0.019±0.003	0.135±0.004	7.438±0.073
S-HY-4	2.530±0.024	0.605±0.005	3.867±0.038	0.024±0.001	0.143±0.002	7.169±0.079
S-HY-5	2.632±0.028	0.623±0.007	4.154±0.047	-	0.152±0.001	7.573±0.091
S-HY-6	2.634±0.025	0.631±0.005	4.428±0.068	0.019±0.003	0.165±0.001	7.873±0.089
S-YC-1	2.888±0.042	0.705±0.007	4.573±0.072	0.027±0.002	0.182±0.003	8.375±0.075
S-YC-2	2.928±0.027	0.715±0.014	4.742±0.074	0.024±0.004	0.216±0.002	8.625±0.086
S-YC-3	2.977±0.072	0.721±0.026	4.870±0.086	0.019±0.001	0.168±0.003	8.754±0.087

S-YC-4	2.924±0.043	0.708±0.007	4.592±0.087	0.022±0.002	0.178±0.004	8.419±0.091
S-YC-5	3.041±0.045	0.714±0.015	4.933±0.064	0.021±0.002	0.189±0.001	8.898±0.091
S-YC-6	3.044±0.036	0.721±0.007	5.258±0.075	-	0.206±0.007	9.239±0.098
S-OD-1	1.367±0.017	0.586±0.007	2.314±0.035	0.010±0.002	0.065±0.007	4.342±0.058
S-OD-2	1.406±0.035	0.546±0.015	2.400±0.053	-	0.078±0.014	4.436±0.058
S-OD-3	1.410±0.046	0.570±0.016	2.464±0.062	0.019±0.002	0.070±0.005	4.527±0.078
S-OD-4	1.445±0.017	0.570±0.005	2.323±0.033	0.021±0.017	0.064±0.018	4.415±0.059
S-OD-5	1.461±0.035	0.575±0.015	2.496±0.046	0.020±0.001	0.061±0.007	4.599±0.076
S-OD-6	1.452±0.014	0.553±0.013	2.660±0.035	0.018±0.003	0.074±0.012	4.748±0.085

(1) SSa, SSc, SSd, SSe, SSf, and SS are the abbreviations of saikosaponin a, saikosaponin c, saikosaponin d, saikosaponin e, saikosaponin f, and saikosaponins, respectively.

(2) N-BT, N-SY, and N-XJ represent *Bupleurum chinense* DC. grown in Baotou city, Sanyuan county, and Xinjiang county, respectively.

(3) S-HY, S-YC, and S-OD represent *Bupleurum scorzonerifolium* Willd. grown in Heyang county, Yuncheng city, and Ordos city, respectively.

Table S2. ANOVA of the effects of variety and growing region on the contents of the five saikosaponins.

Factor	SSa	SSc	SSd	SSe	SSf
Variety	*	*	*	*	*
Growing region	*	*	*	*	*

* significant difference at the level of $p < 0.05$.

Table S3. Comparison of current and reported work

	Present study	Reference 6*
Plants	18 batches of two-year-old North RB and 18 batches of two-year-old South RB, collected from Shaanxi Province, Shanxi Province, and Inner Mongolia Autonomous Region, China.	12 wild North RB and 12 wild South RB, at least one-year-old, collected from the suburban mountainous areas of Beijing, China,
Parts of the plants	Root	Root, stem, leaf, and flower
Extraction method	Sonication in 15% ammonia-methanol	Immersion in 70% aqueous methanol and

	solution and steam distillation	HS-SPME
LC-MS conditions	HPLC-QqQ/MS in MRM and fullscan mode, C18 column, mobile phase: H_2O with 0.1% formic acid (A) and acetonitrile (B)	HPLC-QqQ-LIT/MS in MRM and fullscan mode, C18 column, mobile phase: H_2O (A) and acetonitrile with 0.1% acetic acid (B)
GC-MS conditions	GC-QqQ/MS in SIM and fullscan mode, EI at 70 eV, DB-5MS capillary column, helium at 1.2 mL/min in split mode	EI at 70 eV, DB-5MS capillary column, helium at 1.2 mL/min in splitless mode
Quantitative analysis	Absolute saikosaponin contents	Relative metabolite contents
Qualitative analysis	Tandem MS analysis, retrieval of references and NIST 14 database	Self-complied database
Statistical analysis	HCA, PCA, PLS-DA, ANOVA, T-test	HCA, PCA, OPLS-DA, Venn diagrams, pathway annotation and enrichment analysis
Results and conclusions	1. The absolute content of 5 saikosaponins in Radix Bupleuri from different varieties and regions were obtained.<br>2. Twenty-one saponins and fifty-two volatile oils were identified as the differential compounds.<br>3. The content of saikosaponins was significantly higher in North RB than in South RB.<br>4. There were significant differences in saikosaponins and volatile oils between the two varieties, whether grown in the same region or not.<br>5. The identified differential compounds may be the main active components responsible for the differences in their clinical efficacy.	1. There were significant differences in metabolites between different tissues.<br>2. 144 common differential metabolites were identified between different tissues.<br>3. The content of active components was higher in the root of North RB than in South RB.<br>4. Differential metabolites of the aboveground parts mainly concentrated in monoterpenoid biosynthesis, while those of the root mainly concentrated in sesquiterpenoid and triterpenoid biosynthesis.<br>5. The difference in metabolites and metabolic networks between the two varieties resulted in the difference in the clinical efficacy of Radix Bupleuri.

* Qu, X.; Hu, S.; Li, T.; Zhang, J.; Wang, B.; Liu, C. Metabolomics Analysis Reveals the Differences Between *Bupleurum chinense* DC. and *Bupleurum scorzonerifolium* Willd. *Front. Plant. Sci.* **2022**, *13*, 933849.
